# Dr. Harry Addison Williams

**Published:** 1915-09

**Authors:** 


**Dr. Harry Addison Williams,** Toronto 1908, locum tenens for Dr. Victor Ross of Hamilton, Ont., was shot July 2 by a patient who then killed himself.

